# Study on the characteristic of overburden rock structure and support system of the retraction channel in layered fully mechanized caving face

**DOI:** 10.1038/s41598-024-52015-5

**Published:** 2024-01-16

**Authors:** Weihua Song, Shiqi Yu, Hai Rong, Huice Jiao, Xiaotian Xu

**Affiliations:** 1 College of Mining, Liaoning Technical University, Fuxin, Liaoning China; 2 Shandong Coalfield Geological Planning and Survey Research Institute, Jinan, Shandong China

**Keywords:** Natural hazards, Solid Earth sciences, Materials science

## Abstract

Aiming at the technical problems of frequent dynamic pressure disturbance of regenerated roof and roadway stability control in the process of forming large-section support return channel under the delamination mining, taking 1200-2 fully mechanized caving face of Baiyinhua No. 4 Mine as the engineering background, numerical simulation and theoretical analysis were adopted. The overburden structure of the lower stratified caving face and the loading characteristics of the roof after the expansion are analyzed, and the breaking forms and the limiting conditions of the roof are given.The combined support scheme of anchor rod + metal mesh + steel ladder belt + I-beam insertion + suspension beam + anchor cable + single pillar is studied to form a set of safe and efficient construction support technology system. Practice shows that this scheme can effectively reduce the roof subsidence, narrow the scope of plastic failure zone, and ensure the stability of roadway surrounding rock during the support withdrawal period. Successfully complete the efficient withdrawal of hydraulic support. The research results can provide a good reference for the roof management and the smooth output of coal resources under similar conditions.

## Introduction

In order to ensure the safe and rapid withdrawal of hydraulic support, ensure the smooth replacement of working face and the efficient output of resources, the technology of working face expansion to form a large section support withdrawal channel has been applied to modern mines^1–3^. But after the expansion, the low and hard roof will break, causing the overlying rock to form a cantilever structure^4–6^. Secondary and derivative roof accidents are prone to occur, especially in the fully mechanized caving face with large mining height in the lower layer, which is more prone to roof accidents^7–9^. Therefore, it is particularly important to study a set of safe and efficient timely support scheme for the soft rock roof of the fully mechanized top coal caving face with large mining height after slope expansion. Many scholars have done a lot of targeted research on roof management and slope expansion support of the fully mechanized top coal caving face.

Lv et al.^1^ in response to the deformation law and control problem of surrounding rock in large cross-section excavation under goaf, FLAC numerical simulation was used to analyze the design support scheme. A combined support method of anchor mesh cable, metal shed, and single pillar was proposed, which can effectively reduce roof displacement and achieve good control effect of surrounding rock. Lv et al.^10^ conducted a detailed study and analysis on the plastic deformation and failure characteristics of the surrounding rock of the withdrawal roadway in the fully mechanized top coal caving face of ultra-thick coal seams, based on on-site measurement and numerical simulation experiments. They found that ensuring the stability of the surrounding rock should first ensure the stability of the roof and coal pillar sides. They proposed a joint control scheme of anchor mesh and cable, which achieved efficient moving of the face. Zhang et al.^11^ conducted simulation analysis on the combined support effect of large cross-section retreat tunnels in large mining height working face, and compared it with on-site data and simulation calculation data. Finally, based on the actual situation of tunnel ground pressure, a combined support method is adopted for the tunnel. This creates a new load-bearing system structure for the supported roadway. Feng et al.^12^ used FLAC^3D^ software to simulate the process of layered fully mechanized caving face under thick coal seam slicing mining, compared and analyzed the stress conditions of different monitoring points inside the coal pillars between the main and auxiliary withdrawal channels, and proposed the idea of regional governance for the withdrawal channels. Wang^13^ innovatively proposed the "shield type support retraction process" rapid retraction method, which uses the original hydraulic support of the working face as the shield support. Peng et al.^14^ innovatively proposed a new design method for the withdrawal channel based on the study of mining pressure laws in the mining area, which uses constant resistance large deformation anchor cables and steel strip cross links to support the overall stress in three different stress zones of A, B, and C.

In the study of overlying rock movement of fully mechanized caving face. Ma et al.^15^ based on numerical simulation, similar material simulation, and observation of flushing fluid consumption, identified the failure pattern of soft overlying rocks in layered fully mechanized top coal caving mining of ultra-thick coal seams, with a relatively small range of failure compared to hard and medium hard overlying rocks. Han et al.^16^ used numerical calculation, microseismic monitoring, and transient electromagnetic detection methods to analyze the height of overlying rock failure during the regional layered fully mechanized top coal caving mining process, taking the 55,003 working face of Laohutai Mine as the object, in order to determine the characteristics of overlying rock failure under the conditions of weak overlying rock layered fully mechanized top coal caving mining. The relationship between the mining thickness and the height of overlying rock failure was determined. Liu et al.^17^ used experimental results of similar and numerical simulations to systematically study the fracture process and migration law of overlying strata in fully mechanized top coal caving face with large mining height, and proposed the morphological changes in the evolution of overlying strata fractures. Zhang et al.^18^ analyzed the structural types of overlying rocks in fully mechanized top coal caving face with large mining height, and applied the theory of elastic mechanics to establish mechanical analytical models of masonry beam structure and short cantilever beam structure. The study showed that the direct roof feature has an important impact on the rock pressure behavior, and the multiple key layers of the overlying rock determine the cantilever beam structure formed by the overlying rock, which is the fundamental reason for the strong rock pressure behavior of Baode Mine.

It can be seen from the above literature that some achievements have been made in the research on the law of the expansion support and overlying rock movement. However, the research on the movement characteristics of overlying strata and the manifestation law of mining pressure under the conditions of multiple mining impacts in thick coal seams is not yet clear. Therefore, the research on the support system for expanding the working face under the conditions of fully mechanized mining in thick coal seams needs to be further strengthened. This article takes the 1200-2 working face of Baiyinhua No.4 Mine as the engineering background, According to the actual production situation, the stopping position is 0.6 m ahead of the preset stopping line, causing the expansion position to be within the influence range of the transportation downhill advance support pressure, increasing the risk of roof accidents. A mechanical analysis was conducted on the load-bearing characteristics of the roof under such conditions, and the critical condition for the roof to break was given. A combined support method of anchor rod, metal mesh, steel ladder strip, I-steel inserted beam, suspension beam, anchor cable, and single pillar was proposed. After numerical simulation and underground practice, it was shown that this support scheme can effectively reduce the subsidence of the roof, maintain the stability of the surrounding rock, and facilitate the smooth withdrawal of the hydraulic support The efficient succession of the work face provides strong guarantees.

## Overview of engineering geology

**Figure 1:**
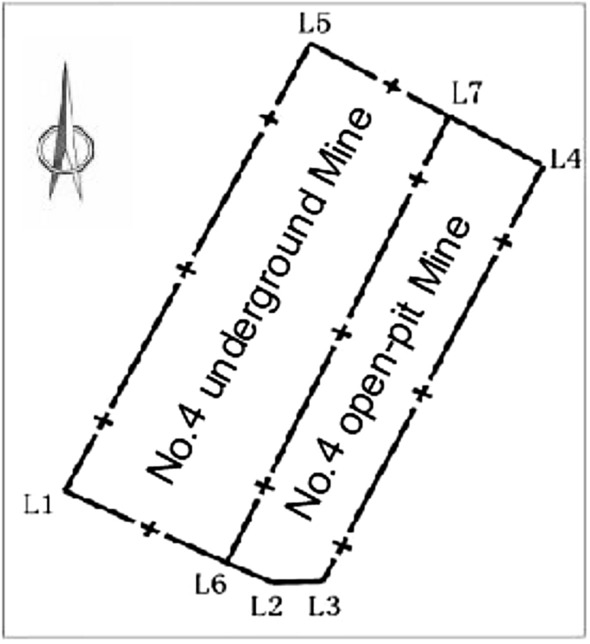
Schematic diagram of the turning point of ore boundary division in the mining area.

Baiyinhua coalfield is located 83 km northeast of Bayanwula Town, West Ujimqin Banner, Xilingol League, Inner Mongolia. The Administrative division belongs to Baiyinhua Sumu and Harigentai Sumu. The mine is located in the middle of the coalfield, surrounded by Baiyinhua No. 1, No. 2, No. 3 and No. 4 open-pit mines respectively. There is no precedent for this kind of mining process surrounded by open-pit mines. Especially when Surface mine is conducted, mining has the strongest impact on land, It will inevitably pose a serious threat to the stability of the roadway during mining.The schematic diagram of the turning point of the boundary division in the mining area is shown in Fig. 1.

1200-2 working face is mined by comprehensive mechanized top coal caving method and adopts strike longwall roadway layout. The 1200-2 fully mechanized top coal caving working face is located in the northeast of the first mining area, with an average mining height of 18 m, a working face length of 221 m, a minable strike of 2421 m, and a working face slope of 7°–13°.Starting from the 1202 return air passage in the east and reaching the 1206 fully mechanized caving face in the west; Starting from the lower section of the first mining area in the south, transport downhill and heading north to the vicinity of exploration line 10.

**Figure 2:**
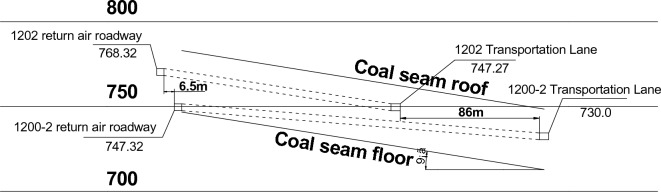
Roadway layer map of working face 1200-2.

The coal seam excavated in the 1200-2 fully mechanized top coal caving face is a coal seam, which is a relatively stable main mining seam that can be mined in the entire area. The average thickness of the coal seam in this area is 36 m, belonging to an extremely thick coal seam. The coal seam is in a monoclinic structure with an inclination angle of 5°–10°.The 1202 working face picks up the upper layer of a coal seam, while the 1200-2 working face picks up the lower layer of a coal seam with a relatively broken regenerated roof. 1200-2 return air channel with an internal displacement of 6.5 m in 1202 return air channel; The horizontal distance between the 1200-2 transportation channel and the 1202 transportation channel is 86.0 m, and the schematic diagram of the roadway layer is shown in Fig. 2.

**Figure 3:**
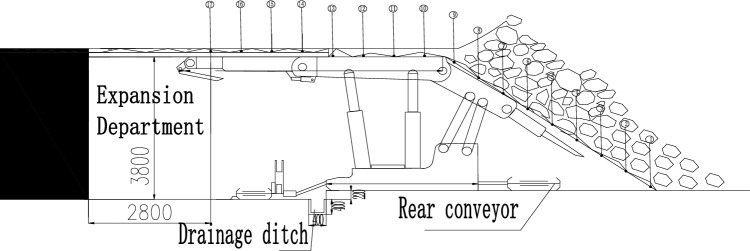
Cross section of side enlargement of working face 1200-2.

The mining of the 1200-2 working face has been completed. In order to meet the space requirements for equipment withdrawal in the 1200-2 working face, it has been decided to expand the cut coal wall. The opening specifications are as follows: after opening, the net width from the front end of the support front beam to the coal wall is 2.8 m, which ensures a net width of 3.25 m from the front end of the front conveyor shovel plate to the coal wall, and a medium height of 3.8 m. The cross-sectional view of the expansion is shown in Fig. 3. In order to determine the support plan for the expansion section, the overlying rock characteristics of the fully mechanized top coal caving face are analyzed to determine the support form and strength.

## Analysis of overlying rock characteristics in fully mechanized top coal caving mining area

### Characteristics of roof structure in fully mechanized caving face

Under the influence of the gravity and mining stress of the overlying strata, the collapsed direct roof and basic roof are re compacted above the layered mining of thick coal seams, forming a regenerated roof.When the lower layer is mined, the regenerated roof rock layer and the collapsed rock layer formed by the top layer mining are affected by repeated mining, and the degree of damage is intensified. Especially under the conditions of fully mechanized top coal caving mining in ultra-thick coal seams, the impact range of high-strength mining activities is large, and the movement range of overlying rock in large goaf areas is large, with intense movement of overlying rock layers, resulting in obvious mining pressure during the mining process. The characteristics of overlying rock fracture movement, overlying rock structure Roof pressure and other issues become more complex as the mining depth increases^19,20^. This complex geological condition will bring huge difficulties to roadway support.

In the process of fully mechanized top coal caving mining, if the thickness is relatively large, the range of surrounding rock movement will inevitably increase. After the top coal is released, the gangue that collapses from the roof cannot fill the goaf in a short period of time, and the basic roof cannot form a stable "masonry beam" hinge structure^21^. The roof rock layer can be divided from bottom to top into the lower direct roof that rises with mining, the upper direct roof of the short cantilever beam structure, and the basic roof that exists in the form of "hinge rock beam"^22–24^. Therefore, the rock layers that affect the stability of the working face roof are the key blocks of the upper direct roof and articulated rock beams that exist in the form of short cantilever beam structures.

### Analysis of roof load after wall expansion

For the 1200-2 fully mechanized top coal caving working face, after advancing to the preset stop mining position, the top coal caving operation will no longer be carried out. The coal wall will be expanded by a combination of manual and coal mining machines. The upper coal will be excavated by hand for 1 m, and the lower 1.8 m of coal will be extracted by the coal mining machine. After the expansion, the length of the cantilever beam formed by the upper direct roof will increase. Due to the stop mining position being one knife ahead of the stop mining line, and the distance of the expansion, Causing the expansion part to be in the area affected by the advanced support of the transportation downhill in the mining area, the upper rock beam is prone to fracture, resulting in roof fall accidents.

**Figure 4:**
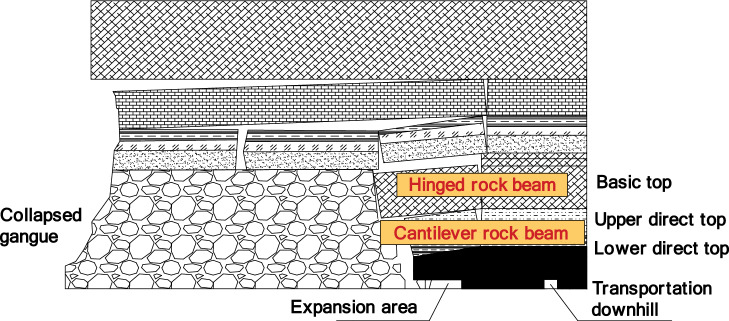
Schematic diagram of cantilever beam + articulated rock beam model.

After the expansion, the top coal is in the system of support and roof action, and the top coal is the medium. The interaction between the excavated roadway and the roof promotes the deformation and damage development of the top coal. The direct roof above the top coal is considered as a cantilever beam that has lost one end of its fixed support. As the length of the cantilever increases, the beam structure will break under the action of self weight load, and the thicker and harder basic roof at the upper level will form an unstable hinged rock beam with the collapsed gangue in the front after the rock beam is directly broken at the lower level, forming sufficient rotational space conditions.Its load will be transmitted to the support and expansion area through the direct roof and top coal. If sufficient support resistance cannot be provided, it will lead to frame compression or roof fall accidents,Therefore, it is necessary to determine the breaking step distance of the roof rock beam and calculate the support reaction force at the fixed end of the cantilever beam, including horizontal force, vertical force, and bending moment, based on the force balance condition.The schematic diagram of the cantilever beam + articulated rock beam model is shown in Fig. 4.

The load-bearing characteristic of this model is that when the cantilever beam loses stability alone, it will generate small periodic pressure. If the upper "articulated rock beam" has sufficient rotational space, it will cause the "articulated rock beam + cantilever beam" to lose stability simultaneously, resulting in large periodic pressure. Therefore, the working resistance of the support design must be greater than the pressure generated by the simultaneous instability of the "composite beam".Due to the high mining height of the fully mechanized top coal caving face, the direct roof fracture, roof cutting and sliding occur, causing the basic roof and overlying collapse zone to sink accordingly. The support rapidly increases resistance, quickly reaching the rated working resistance of the support. One end is pressed on top of the support, and the other end is pressed on the middle coal body^25^. Under this stress condition, the support is in a constant load state, and the working resistance of the support depends on the quality of the collapsed overlying rock. The height of the collapsed zone can be calculated using formula (1), and then the stress analysis of the roof collapsed rock beam can be carried out:

1 $${H}_{m}=\frac{100\sum M}{4.7\sum M+19}+2.2$$

In the equation, H_m_, the height of the collapse zone, m; $\sum M$ is the cumulative mining thickness, m; For fully mechanized top coal caving mining, the total height of coal cutting and caving,

The average pressure of the collapsed roof can be obtained:

2 $${k}_{1}{q}_{1}={H}_{m}{\gamma }_{m}$$

**Figure 5:**
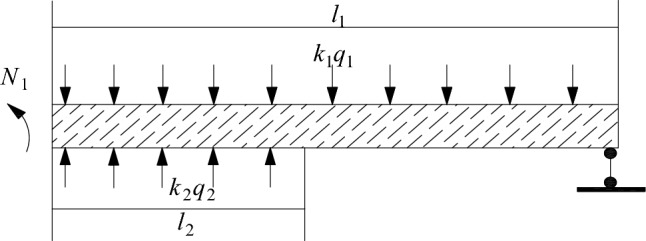
Mechanical analysis diagram of roof loading.

In the equation, ${\gamma }_{m}$ is the bulk density of the rock mass,

According to Fig. 5, the load-bearing mechanics analysis of the fractured roof and the collapsed overlying rock can be used to calculate the pressure F on the support and the bending moment N_1_ acting on the moving boundary of the rock layer:

3 $$F=\frac{{k}_{1}{q}_{1}{l}_{1}^{2}d}{{2l}_{1}-{l}_{2}}$$

4 $${N}_{1}=\frac{1}{2}{l}_{1}^{2}{k}_{1}{q}_{1}$$

At the fracture line, the bearing capacity of the support is a constant load condition. If the calculated working resistance of the support at this stage and the resistance F_S_ provided by the expansion support plan meet:

$${{\text{F}}}_{{\text{S}}} > {\text{F}}$$

The support scheme meets the design requirements and significantly reduces the risk of large-scale roof fall and frame compression accidents^26,27^.

### Critical conditions for roof breaking

**Figure 6:**
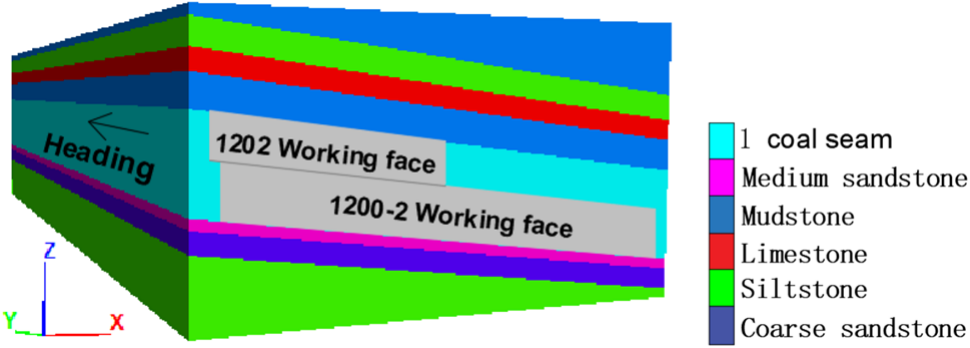
FLAC^3D^ numerical model.

In order to determine the breaking distance of the roof cantilever structure, based on the actual production situation, the finite element numerical simulation software FLAC^3D^ was used to simulate the mining of the working face and observe the collapse of the overlying rock. According to the roadway layout of the working face, establish a numerical model for mining the working face, as shown in Fig. 6, selecting the Mohr Coulomb constitutive modelwith a model size of 500 m × 200 m × 110 m (length × wide × High), a total of 405,600 units have been established.

The mechanical parameters of each coal layer are shown in Table 1. The boundary conditions of the calculation model are determined as follows:

1. Apply constraints along the X-axis to the boundaries at both ends of the X-axis of the model, that is, the displacement in the X-direction of the boundary is zero;
2. Apply constraints along the Z-axis to the boundaries at both ends of the Z-axis of the model, that is, the displacement in the Z-direction of the boundary is zero;
3. The Y-axis (bottom) boundary of the model is constrained and fixed along the Y-axis, meaning that the displacement in the Y-direction of the bottom boundary is zero;
4. The top of the model is a free boundary.

**Table 1:** Physical and mechanical parameters of coal seam, roof and floor in working face.

Rock name	Density/kg m^−3^	Tensile strength/MPa	Bulk modulus/MPa	Shear modulus/MPa	Cohesion/MPa	Internal friction angle/°
Siltstone	2601	1.7	9200	6600	5.3	36
Coal	1267	0.7	4400	2800	1.6	30
Mudstone	2668	0.9	8600	7700	2.4	32
Coarse sandstone	2719	1.5	8500	7500	4.5	33
Medium sandstone	2719	2.1	6300	3580	5.2	35
limestone	2610	1.7	7800	6500	4.5	34

**Figure 7:**
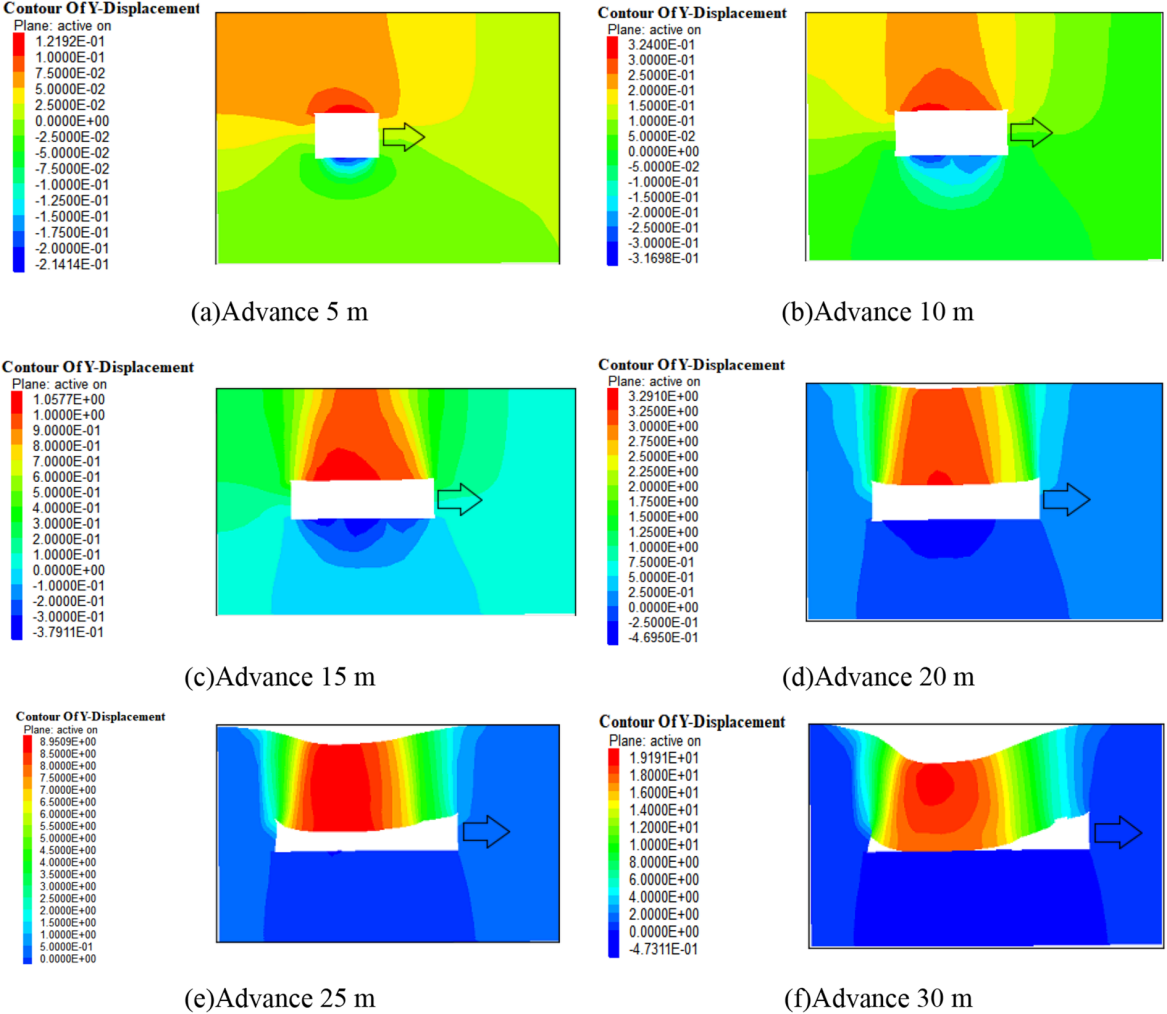
Vertical displacement nephogram of surrounding rock.

**Figure 8:**
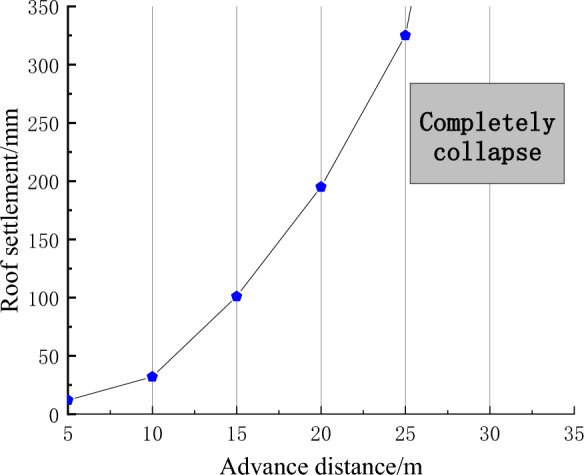
Roof subsidence during mining in 1202-2 working face.

According to the numerical simulation results, after the formation of the goaf, the overlying rock layer will gradually sink until it completely collapses under the influence of the gradually increasing goaf and self weight stress. The vertical displacement cloud map of the surrounding rock towards different advancing distances is shown in Fig. 7, and the subsidence amount of the roof and the advancing distance are shown in Fig. 8. After 15 m, the subsidence amount of the roof gradually increases, and it increases to 325 mm at 25 m. When mining to 30 m, the roof completely collapses. Therefore, it is inferred that the initial pressure step distance of the 1200-2 working face is about 30 m.

### Stability analysis of regenerated roof considering time effect

Through the above simulation, it can be seen that the upper layer of backfilling will inevitably cause the collapse and re compaction stage of the roof. Only after forming a stable regenerated roof can the lower layer of backfilling be carried out. The formation of a regenerated roof with a certain strength and stability by the collapsed gangue is completed by two stages: compaction and consolidation. Compaction is the process of breaking the regenerated gangue, reducing its volume, porosity, and stabilizing the pressure under the pressure of the overlying rock layer; Compaction refers to the transformation of regenerated gangue from loose to solid, generating a certain degree of cohesion and strength. Compaction occurs before compaction, and the greater the movement pressure of the overlying rock layer and the greater the volume shrinkage after compaction, the higher the strength after compaction. The longer the compaction time, the higher the strength and stability of the regenerated roof. The critical time T required for the formation of a regenerated roof with a certain strength stability by the falling gangue should be determined by the compaction time t_1_ and the time t_2_ required to reach a strength not lower than the critical strength (≥ 1.1 MPa) after compaction, is:

5 $$ {\text{T}} \ge {\text{t}}_{{1}} + {\text{t}}_{{2}} $$

In the formula: t_1_—is the compaction stability time of the regenerated rock block; t_2_—the pressing time after which the critical strength torsion (≥ 1.1 MPa) is reached.

The compaction process of regenerated gangue is not only related to the size of its own gangue particles, but mainly depends on the thickness, compaction step distance, and strength of the overlying moving rock layer. During the research process, a force gauge was used to monitor the pressure changes of the support bracket behind the coal wall of the advancing mining face, in order to determine the range within which the falling gangue reaches a stable pressure, and indirectly obtain the compaction time t_1_ value.

The movement of the rear roof of the mining face has a clear distribution pattern of "three zones", namely the pressure relief zone, active zone, and stable zone. According to the actual production data on site, the range of the pressure relief zone behind the working face is 0–15 m, and the range of the active zone is 15–100 m. After 100 m, it is a relatively stable pressure zone, indicating that the fallen gangue in the goaf has been basically compacted and the roof is tending to be stable. Therefore:

6 $$ {\text{t}}_{{1}} = {\text{L}}/{\text{V}} $$

In the formula, L—represents the stable pressure distance behind the working face, m; V—the average pushing speed of the V—working face, m/day.

For the 1202 working face of Jinggong Mine, if L = 100 m and V = 2.5 m/d, then t_1_ = 100/2.5 = 40 days.

**Figure 9:**
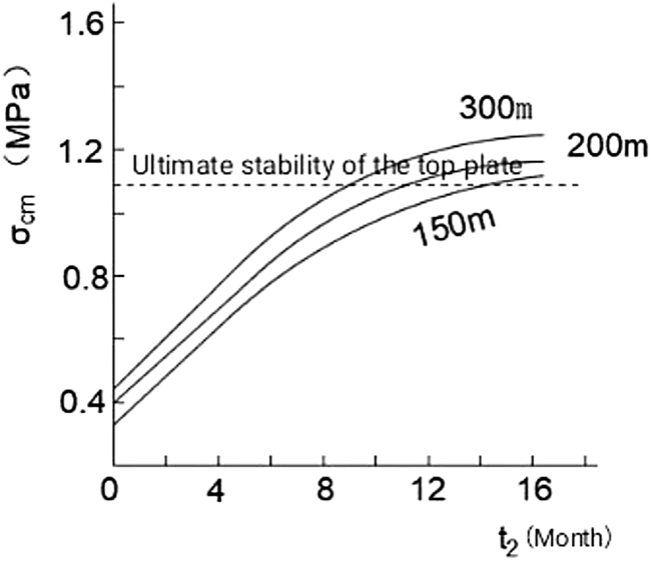
Diagram of ultimate strength of regenerated roof with time at different mining depths.

For the regenerated roof formed by different mining depths, the t_2_ value can be directly used in the Fig. 9. Based on the ${\upsigma }_{{\text{cm}}}$ = $\mathrm{\varnothing }$(t_2_) curve and the critical strength ${\upsigma }_{{\text{cm}}}$ ≥ 1.1 IMPa, the critical time t_2_ value for forming a stable regenerated roof with a strength not less than 1.1 MPa can be obtained.

For the 1202 working face of Jinggong Mine, its mining depth is about 300 m. According to the graph, when t_2_ is ≥ 8.5 months, it is ${\upsigma }_{{\text{cm}}}$ ≥ 1.1 MPa.

Then T ≥ t_1_ + t_2_.

40/30 + 8.5 = September August.

Therefore, the regeneration time of the regenerated roof after mining at the 1202 working face of Jinggong Mine should not be less than 9.8 months. Therefore, during downward mining, the 1200-2 working face should be mined 9.8 months after the 1202 working face is mined.

## Study on the support system of the expansion area

### Support scheme design

In response to the mechanical loading characteristics of the roof of the fully mechanized caving working face mentioned above and the problem of relatively broken roof of the coal roadway in the 1200-2 working face, a joint support scheme with anchor bolts and cables as the core is proposed. The suspension theory and composite beam theory are comprehensively used to calculate the parameters of the anchor bolts and cables. After determining the support scheme, the limit equilibrium plastic zone method is used to verify the support parameters.

1. Anchor bolt parameter verification
  ① Checking calculation of anchor rod length
    The length L of the anchor rod is composed of the length L_1_ of the anchor rod anchoring section, the exposed length L_2_ of the anchor rod, and the effective length L_3_ of the anchor rod. The exposed length L_2_ of the anchor rod is composed of the thickness of the base plate, the thickness of the nut, and the allowance, generally taken as 0.1 m.
    7 $$ L = L_{1} + L_{2} + L_{3} $$
    In the equation: $L$—Anchor rod length, m; $L_{1}$—The depth of anchor rod anchoring into stable rock layers can generally be taken as 0.2 m based on experience; $L_{2}$—The exposed length of the anchor rod in the tunnel is taken as 0.1 m; $L_{3}$—Effective length of anchor rod, m;
    The effective length L_3_ of the anchor rod should be greater than the range H of the loose and fractured zone of the surrounding rock. As the roof of the tunnel is a coal seam, the solidity coefficient is less than 2. H can be estimated using the parabolic pressure arch theory:
    8 $$ H = \frac{1}{f}\left[ {\frac{B}{2} + h\cot \left( {45^\circ + \frac{\varphi }{2}} \right)} \right] $$
    In the equation: B—Tunnel width, taken as 2.9 m; h—Tunnel excavation height, 3.8 m; f—The Proctor's rock firmness coefficient is taken as f = 1.9 based on the rock firmness classification table.
    φ—Internal friction angle, °; After calculation, it can be concluded that the pressure arch height H is 1.87 m, and the minimum length L of the anchor rod is 2.16 m.
  ② Diameter of anchor rod body
    The diameter of the anchor rod body is determined based on the strength principles of the bearing capacity and anchoring force of the rod body, as follows:
    9 $$ d = 35.52\sqrt {\frac{Q}{{\sigma_{t} }}} $$
    In the equation: d—Diameter of anchor rod body, mm; Q—Anchor rod anchoring force, 125 kN; $\sigma_{t}$—The tensile strength of the rod material, 570 MPa.
    After calculation, the diameter of the anchor rod should be at least 16.63 mm.
  ③ Verification calculation of anchor rod spacing and row spacing
    10 $$ a = \left( {Q/kH\gamma } \right)^{1/2} $$
    In the equation: a—The spacing and row spacing of anchor rods, m; Q—The design anchoring force of the anchor rod, 125 kN; k—Safety factor, k = 2; H—Pressure arch height, 1.87 m; γ—The bulk density of falling coal and rock, 12.8 kN/m^3^.
    After calculation, the maximum spacing between anchor rods is 1.62 m.
2. Checking calculation of anchor cable parameters
  ① Calculation of anchor cable length inspection parameters
    11 $$ L = L_{a} + L_{b} + L_{c} + L_{d} $$
    L—Total length of anchor cable, m; $L_{a}$—The anchoring length of the anchor cable penetrating into the stable rock layer is taken as 1.5 m; $L_{b}$—The thickness of unstable rock layers that need to be suspended, 2.87 m; $L_{c}$—The thickness of the upper tray and anchorage, taken as 0.2 m; $L_{d}$—The exposed tension length is taken as 0.3 m;
    After calculation, the minimum length of the anchor cable is 4.87 m.
  ② Checking calculation of anchor cable spacing
    12 $$ D \le {{nq_{s} } \mathord{\left/ {\vphantom {{nq_{s} } {k^{\prime}BH\gamma }}} \right. \kern-0pt} {k^{\prime}BH\gamma }} $$
    In the equation: $n$—The number of anchor cables in each row, taken as 2; $k^{\prime}$—Safety factor, taken as 2; $q_{s}$—The ultimate breaking force of a single anchor cable, taken as 220 kN; B—The maximum caving width of the roadway is 2.9 m; H—Falling height, 2.35 m; γ—The bulk density of falling coal and rock, 12.8 kN/m^3^.
    After calculation, the spacing between anchor cables should be less than 2.52 m.

Based on the above calculation results, the layout of anchor rods, cables, and individual pillars is continuously optimized. With a certain margin coefficient left, it is decided to adopt a combined support method of anchor rods, metal mesh, steel ladder belts, I-steel inserted beams (two per beam), suspension beams, anchor cables, and individual pillars. The specific parameters are: the top anchor rod adopts Φ twenty × 2200 mm full thread equal strength anchor rod with a spacing of 900 × 750 mm, with 3 anchor rods per row; The top plate beam is made of 12 # I-beam with a length of 5.5 m. Two I-beam beams are inserted above the top beam of each bracket. Two anchor cable holes are arranged near the opening side beam head of the I-beam, with a spacing of 1000 mm. The distance between the top hole and the beam head is 300 mm, and two I-beam beams are injected for each I-beam Φ seventeen point eight × 8000 mm steel strand; Inject DW-2.5 monomer at the end of each inserted beam near the coal slope side beam, with a column spacing of 750 mm. Additionally, install a row of inclined I-steel suspension beams at a distance of 0.5 m from the coal slope, with a length of 4.5 m. Each beam is equipped with 5 anchor cable holes with a spacing of 1000 mm Φ seventeen point eight × 8000 mm steel strand anchor cable.

**Figure 10:**
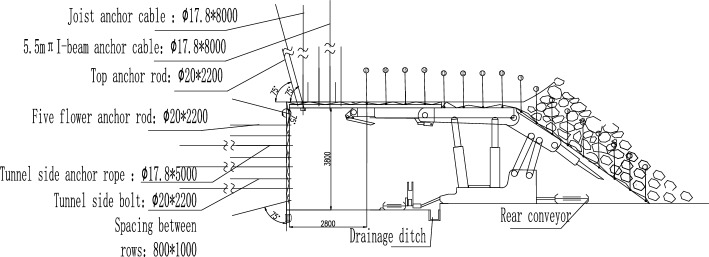
Cutaway support profile.

**Figure 11:**
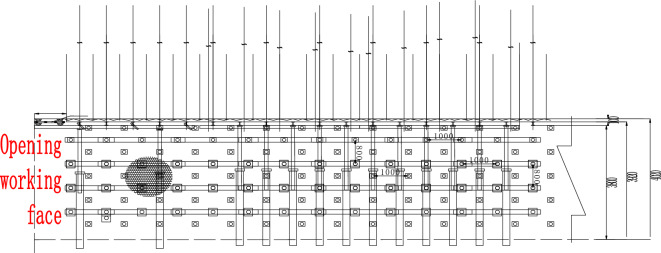
Top view of side opening support.

The support adopts a combination of anchor rods, metal mesh, and short anchor cables (self-made steel wire ropes and belts) for support. The use of auxiliary anchor rods Φ twenty × 2200 mm full thread equal strength anchor rod with a spacing of 800 rows × 1000 mm; The use of auxiliary anchor cables Φ seventeen point eight × 5000 mm steel strand with a spacing of 800 rows × 1000 mm, each anchor cable and anchor rod form a "five flower" arrangement. The support plan for the opening is shown in Figs. 10 and 11.

Below, the limit equilibrium plastic zone method is used to verify the parameters of the proposed support scheme.

1. Plastic zone radius under limit equilibrium
  13 $$ R_{s} = R_{0} \left[ {\frac{{\left( {1 - \sin \phi } \right) \times \left( {K\gamma H + C \times \cot \phi } \right)}}{C \times \cot \phi }} \right]^{{\frac{1 - \sin \phi }{{2\sin \phi }}}} $$
  In the equation: *R*_*s*_—The radius of the plastic zone in the roadway, m; $R_{0}$—The radius of the outer circle of the roadway, 4.48 m; $\gamma$—The bulk density of falling coal and rock, 12.8 kN/m^3^. $H$—Tunnel burial depth, 286 m; $C$—Cohesive force of surrounding rock, 0.6 MPa; $\varphi$—Internal friction angle of surrounding rock, 32°; K—Stress concentration coefficient, taken as 2.5;
  After calculation, the radius of the plastic zone in the roadway of the expansion section has been determined $R_{s} = 9.09$ m.
2. Calculate the support force required to maintain the rock in the limit equilibrium zone from falling.
  The thickness of the top rock load is:
  14 $$ h_{d} = R_{s} - \frac{b}{2} $$
  In the equation: $R_{s}$—Radius of plastic zone in roadway, m; $b$—Tunnel height, 3.8 m; After calculation:$h_{d} = 7.19$ m.
  The minimum support force required to maintain the rock in the limit equilibrium zone from falling  is:15
  In the equation: $\gamma_{i}$—Unit weight of layer i; $h_{i}$—The thickness of the i-th layer.
  After calculation, the minimum support force is 92.03 kN/m.
  After the expansion, the distance between the hydraulic support and the front coal wall is 2.9 m, so the minimum support force to maintain the rock in the limit equilibrium zone in front of the hydraulic support within a width of 1 m is 266.89 kN.
3. Support resistance provided by anchor cables:
  16 $$ P_{s} = n\frac{{q_{s} }}{B \times D}. $$
  In the equation: *q*_*s*_—The breaking force of the anchor cable is 250 kN for a 17.8 mm anchor cable; *D*—Anchor cable spacing, with an anchor cable spacing of 1.5 m; *n*—The number of anchor cables in each row, taken as 2; B—Tunnel width, 2.9 m; After calculation, ${p}_{s}$ = 111.49 kN/m^2^.
  The support resistance provided by the 1 m wide anchor cable from the front of the hydraulic support to the coal wall after opening is 323.32 kN.
4. Support resistance provided by anchor rods
  17 $$ P{}_{m} = \eta \cdot q{}_{m}/D_{m}^{2} $$
  In the equation: *q*_*m*_—The anchoring force of the anchor rod is 125 kN; $D_{m}^{2}$—Anchor rod spacing and row spacing, taken as 0.9 × 1 m; *η*—Anchor rod support coefficient, 0.35; After calculation: ${p}_{m}$=48.61 kN/m^2^.
  The support resistance provided by the 1m wide anchor rod from the front of the hydraulic support to the rear of the coal wall after excavation is 140.97 kN.
5. Total support resistance
  The total support resistance provided by the anchor rod and cable in front of the hydraulic support is 464.3 kN.
6. Support safety factor
  The safety factor K is the ratio of total resistance to required support force, which is 1.74, meeting the requirement of a safety factor of not less than 1.5.
  The safety factor is the ratio of total resistance to required support force, which is 1.78, meeting the requirement of a safety factor of not less than 1.5. The support parameters all comply with the calculation results, and the support strength can meet the requirements of roadway excavation support.

### Numerical simulation analysis of support schemes

In order to verify the effectiveness of the above support scheme, numerical simulation and comparison were conducted on the deformation of the surrounding rock of the roadway at the opening of the 1200 2 working face with and without support, In FLAC^3D^ software, there is a built-in Cable structural unit that can generate shear resistance along its length direction, thereby simulating the mechanical form of anchor rods and anchoring agents in surrounding rock. The mechanical properties of the cable structural unit are mainly related to factors such as its geometric shape, material, anchoring agent performance, and pre tightening force. This parameter can be measured through laboratory experiments. A row of single pillars near the coal wall are simulated using Beam structural units, and the hydraulic support on the right is equivalent to a single pillar of two rows of Beam structure. The model size is 60 m × 30 m × 50 m (length × wide × High), a total of 76,250 units have been established.

**Figure 12:**
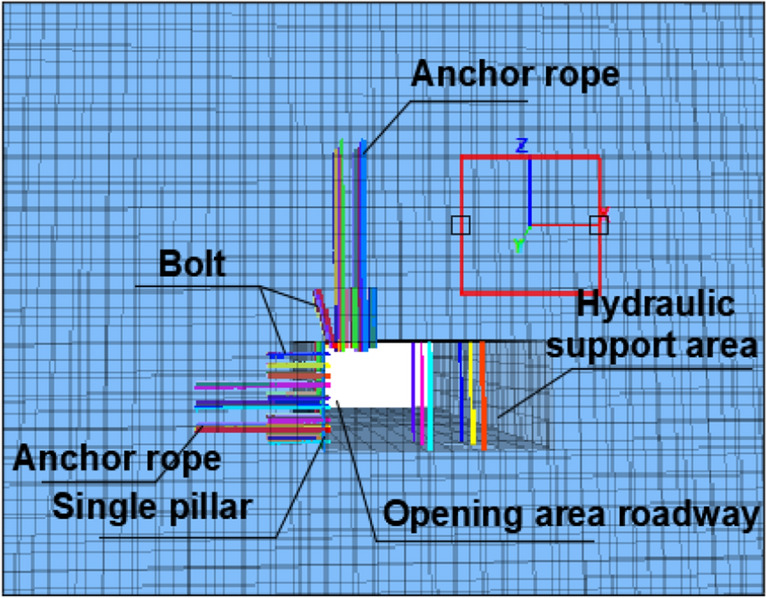
FLAC^3D^ numerical model.

The FLAC^3D^ numerical model is shown in Fig. 12, and the mechanical parameters of the coal and rock layers are shown in Table 1.

**Figure 13:**
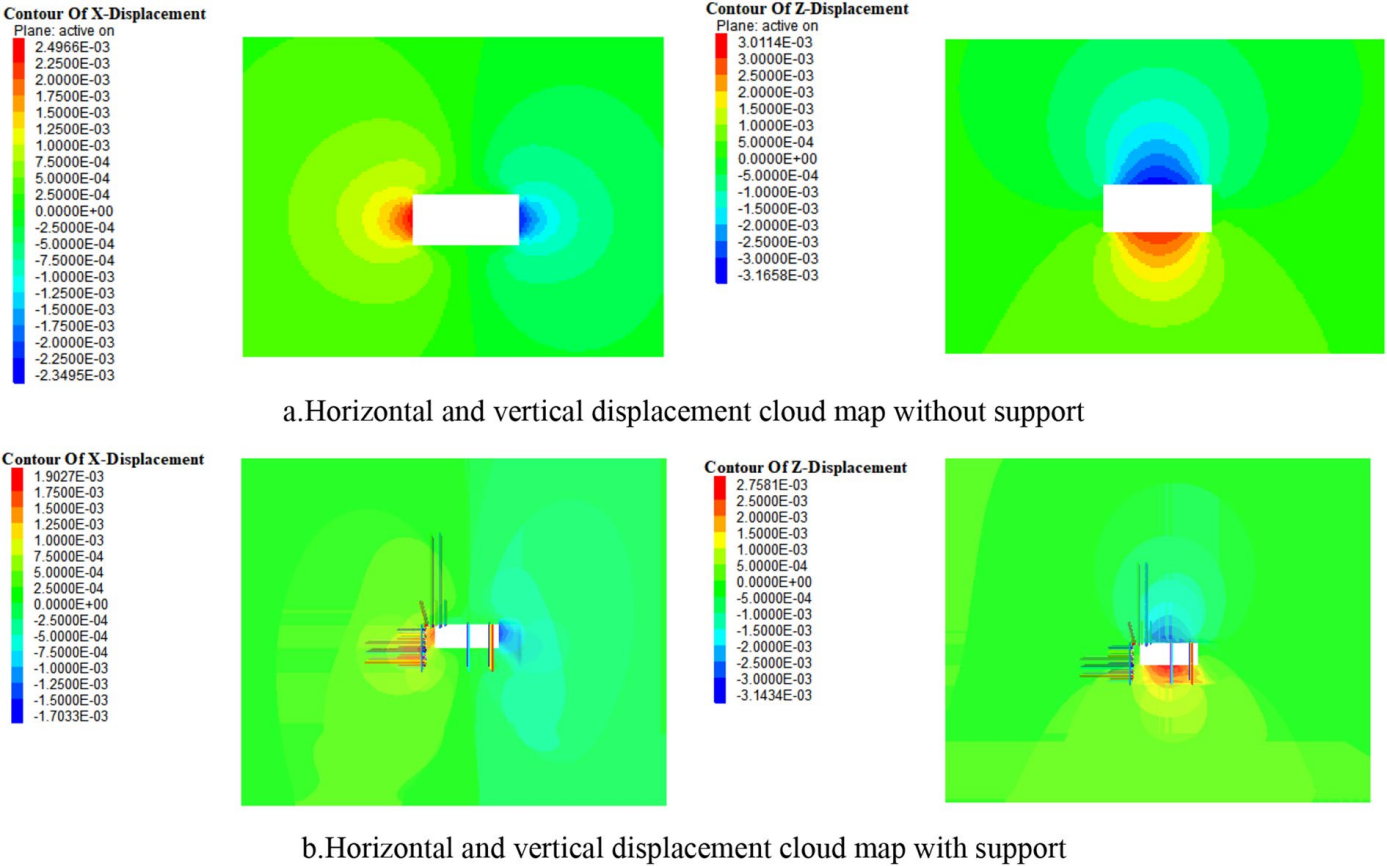
Displacement nephogram comparison.

**Figure 14:**
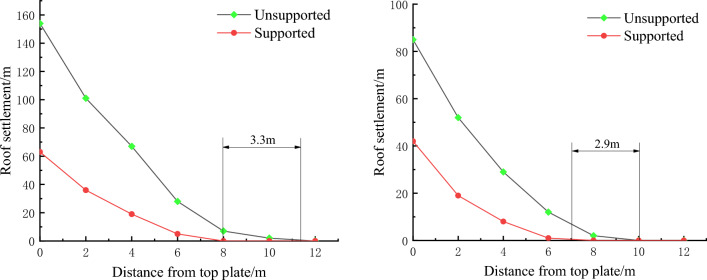
Comparison diagram of roof and coal side displacement.

The displacement cloud map of the roof and coal slope under different support conditions is shown in Fig. 13, and the comparison of displacement is shown in Fig. 14. According to the simulation results, the maximum deformation of the roadway and roof without support is 85 mm and 154 mm, respectively. When using this support scheme, the maximum deformation of the roadway and roof is 42 mm and 63 mm, respectively, with a decrease of 50.5% and 59.1%, respectively.When the displacement is 0, according to the distance from the roof and coal wall, the plastic failure zone of the roof and roadway with support is reduced by 3.3 m and 2.9 m respectively compared to the plastic failure zone without support. It can be seen that this support scheme can effectively reduce the subsidence of the roof, suppress the development of surrounding rock cracks, reduce the range of plastic failure zone, and maintain the stability of the surrounding rock of the roadway during excavation.

## Engineering practice verification

**Figure 15:**
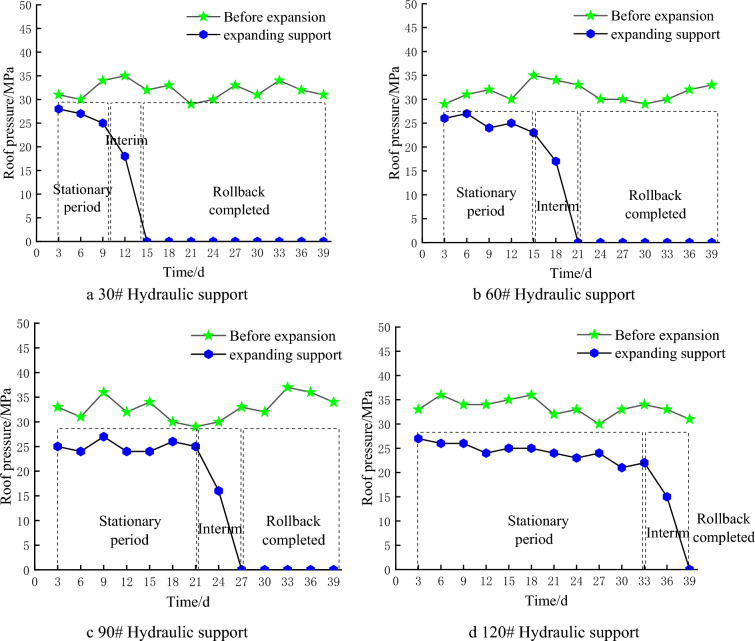
Top plate stress monitoring diagram.

The above support scheme was applied to the excavation and wall expansion support project of the 1200-2 working face. In order to test the actual support effect, four equidistant hydraulic supports were selected to observe and compare the stress data of the roof monitoring before and within 40 days after the wall expansion support. The comparison of roof stress monitoring is shown in Fig. 15. According to monitoring data, after using the above support scheme to form the roadway, the stress of the roof has been significantly reduced. Before the support is withdrawn, the roof pressure will be in a stable period of around 25 MPa. When the support is withdrawn close to the monitored support, there will be a transition period of significant decrease in the roof pressure of the monitored support due to the withdrawal of the front support, until it is safely withdrawn.

Within 40 days of the support retraction, there were no serious roof fall or wall collapse accidents, indicating that the above support plan can effectively reduce or transfer roof stress, reduce roof subsidence, and maintain the stability of the surrounding rock.

## Conclusion

1. Analyzed the characteristics of the overlying rock structure of the fully mechanized top coal caving face under thick coal seams, and aimed at the roof support problem of the retreat channel formed by the expansion of the fully mechanized top coal caving face. Through theoretical analysis, it was found that the roof of the 1200-2 fully mechanized top coal caving face can be divided into a lower level direct roof that follows mining, a upper level direct roof with a short cantilever beam structure, and a basic roof that exists in the form of a "hinged rock beam" from bottom to top.
2. Through numerical simulation and Theoretical Mechanics analysis, the first caving step of 1200-2 fully mechanized top coal caving face and the roof breaking form after the slope expansion are determined, and the critical conditions for large-scale roof fall and frame pressing accidents are given.
3. Based on the surrounding rock control technology, a combined support method of anchor rod, metal mesh, steel ladder and strip, I-beam insertion beam, suspension beam, anchor cable, and single pillar is developed. Qualitative analysis was conducted using numerical simulation. The simulation results showed that compared to no support conditions, using the design scheme for support reduced the maximum subsidence of the roof and coal slope in the support area by 59.1% and 50.5%, respectively. The plastic failure area of the roof and roadway slope decreased by 3.3 m and 2.9 m, respectively.
4. By using the limit equilibrium method, suspension theory, and composite beam theory, the design scheme was verified and the adaptability of the design support scheme to the tunnel cross-section was determined after the excavation of the 1200-2 working face. It was confirmed that the support resistance of anchor rods and cables in the design support scheme can meet the load of rock layers in the plastic zone. The on-site engineering application shows that this support scheme can effectively reduce the stress of the roof and ensure the stability of the surrounding rock.

## Data Availability

The data used to support the findings of this study are available from the corresponding author upon request.
